# Wideband acoustic immittance in early childhood: The role of outer and middle ear in auditory development

**DOI:** 10.1016/j.clinsp.2025.100841

**Published:** 2025-11-19

**Authors:** Ana Paula Bruner, Sumitrajit Dhar, Uzma Akhtar, Alessandra Spada Durante, Renata Mota Mamede Carvallo

**Affiliations:** a Faculdade de Medicina da Universidade de São Paulo (FMUSP), São Paulo, SP, Brazil; b Northwestern University, Evanston, USA; c Rush University Medical Center, Chicago, USA; d Universidade Federal de São Paulo (Unifesp), São Paulo, SP, Brazil; e Faculdade de Ciências Médicas da Santa Casa de São Paulo (FCMSCSP), São Paulo, SP, Brazil

**Keywords:** Middle ear, Infant, Child, Hearing tests, Acoustic impedance tests

## Abstract

**Objective:**

To investigate essential properties of acoustic transmission, considering the influence of anatomical and functional changes on the development of the auditory system.

**Methods:**

This is a cross-sectional observational study. Five groups were established depending on the age range of 117 normal-hearing children: 35 Newborns (NB), 15 infants aged six to eight months (6‒8 m), 10 children aged three to five years (3‒5y), 28 aged six to eight (6‒8y) and 29 aged nine to eleven years (9‒11y). Wideband Acoustic Immittance (WAI) data were recorded using a Titan tympanometer (Interacoustics®).

**Results:**

The highest absorbance measurements were found in NB and 6‒8 m groups than in children aged three years and older – groups 3‒5y, 6‒8y and 9‒11y – whose measurements were quite similar to each other, being higher in the range of 2k to 6k Hz. Therefore, unlike the reduction in absorbance at low frequencies, which should occur after the first month, at high frequencies, it seems to occur only after the first six months of life. Resonance Frequency (RF) data showed a significant increase with age. In newborns, RF was lower than in infants (6‒8 m), and there were no apparent changes from the age group 3‒5y to 9‒11y.

**Conclusion:**

Anatomical and physiological changes in the outer and middle ear throughout childhood lead to changes in WAI measurements, such as equivalent ear canal volume and compensated acoustic admittance increased throughout childhood, while resonance frequency only increased in the first three years of life. The variable pattern of acoustic absorbance is likely representing the various structural changes throughout childhood that affect mass and stiffness components independently.

## Highlights

• ECV, Y_TM_, absorbance and RF of healthy ears change throughout childhood.
• In newborns, low-frequencies absorbance and RF are attributed to EAC wall mobility.
• ECV, absorbance and RF change notably before 6-months and stabilize after 3-years.
• Absorbance decreases at 0.25‒0.6k Hz up to 6-months, then decreases at 2k‒6k Hz.
• From birth to preadolescence, specific WAI patterns must be considered.

## Introduction

The maturation of the human auditory system depends on a series of anatomical, physiological, and functional changes. A combination of structural modifications – genetically programmed and environmentally influenced – shapes the development of auditory mechanisms essential for communication.^1^, ^2^, ^3^, ^4^

At the most peripheral level, the External Ear (EE) and Middle Ear (ME) are not fully mature at birth. The External Auditory Canal (EAC), shorter and straighter in newborns, gradually changes orientation, diameter, and length as the skull grows.^5^^,^^6^ Meanwhile, the more compliant canal walls progressively thicken and stiffen with ossification.^4^^,^^6^, ^7^, ^8^ These changes mainly occur until about three years of age,^5^^,^^6^ the same period in which the Tympanic Membrane (TM), which is nearly horizontal at birth, becomes thinner and more vertical.^5^^,^^7^ Additionally, changes in the distribution of fibers, three-dimensional curvature, and rigidity of the TM can extend until the end of adolescence. The middle ear cavity also enlarges,^1^^,^^6^ especially in height and the distance between the TM and the footplate stapes, which can influence the orientation of the ossicles. Postnatal changes related to ossification and increased weight and size of the ossicles are subtle, but there is substantial stiffening of the ossicular joints up to six months, due to the absorption of mesenchyme adhered to the ossicular chain.^1^^,^^5^

Postnatal maturation of the conductive pathway, therefore, can modify the acoustic properties of the outer and middle ears^3^ influencing energy transmission. That is, anatomical and physiological variations related to growth can lead to changes in energy propagation, mechanical efficiency, filtering and resonance, and transmission delays.^2^^,^^9^

Traditionally, the middle ear’s impedance and admittance properties are characterized using single-frequency tympanometry. More recently, Wideband Acoustic Immittance (WAI) has been used to fully characterize the middle ear’s response across a wide range of frequencies. WAI refers to a set of functional measurements obtained in response to stimuli such as clicks, chirps, or simultaneous pure tones. By comparing the incident sound pressure with that reflected by the TM, acoustic absorbance or reflectance measurements are quantified for each frequency.^10^

Admittance and absorbance are complementary functions of acoustic transfer and vary depending on the frequency of the incident sound pressure.^2^ The acoustic-mechanical models of outer and middle ear systems include both natural stiffness, mass, and resistance components, many of which are known to develop during the first six months of human life and mature by early adolescence. Furthermore, the interaction between mass reactance and stiffness determines the transfer function of the conductive pathway. The Resonance Frequency (RF) corresponds to the frequency transmitted most efficiently by the system where mass reactance and stiffness are perfectly counterposed, canceling each other out. At resonance, there is maximum transmission in direct and reverse transfer, due to the reversal in phase and with a greater amplitude of tympanic-ossicular displacement.^10^, ^11^, ^12^

WAI provides an in-depth view of the state and physical properties of the middle ear, providing insight into how energy transmission occurs in all frequency ranges important to human hearing.^10^^,^^13^ Because they are less influenced by the EAC and probe positioning, they have been increasingly used in clinical research,^4^^,^^8^, ^9^, ^10^ allowing the differentiation of conductive disorders based on their typical absorbance curves.^9^^,^^10^^,^^13^

Several experiments with acoustic immittance measures have shown considerable changes throughout childhood.^4^^,^^8^^,^^9^^,^^14^, ^15^, ^16^ The Ear Canal Volume (ECV) may be approximately three times higher in adults than in infants.^17^^,^^18^ The acoustic admittance compensated (Y_TM_) also varies during growth, as it is related to the volume of ME cavity. In infants aged one to 24-months, it corresponds to approximately half of that found in the adult ear.^6^

Absorbance measurements also vary throughout life, with the measured changes starting at low frequencies and moving to high frequencies.^3^^,^^16^^,^^19^ Due to the vibration of the ME and the resonance of the OE and ME, the frequencies between 1 kHz and 4 kHz are transmitted more efficiently. However, the ears of older infants still exhibit higher levels of absorbance at low frequencies than, like seven-year-olds, when values close to those of adults are reached.^8^ At high frequencies, levels similar to those of adults are reached at age 12.^3^^,^^19^^,^^20^

Therefore, there is a consensus on the advantages of WAI, particularly in children, due to the speed in obtaining relevant data that contribute to the diagnosis of middle ear alterations,^13^^,^^20^, ^21^, ^22^ but its effectiveness in diagnosing conductive alterations with high sensitivity and specificity still depends on the use of different normative criteria in the interpretation of measurements according to the equipment and the age of the individual.^4^^,^^9^^,^^10^^,^^13^^,^^15^^,^^16^

Despite the great variability in RF, it is known that it depends on the most prominent component of the system's susceptance, either mass or stiffness.^9^^,^^12^^,^^21^ In newborns and infants, the RF is usually low, attributed to the EAC, as it would result from the preponderant influence of the mass component in the cartilaginous tissue of the EAC.^8^^,^^11^^,^^12^^,^^15^ In the ME, the RF in children is usually close to that of adults, with greater acoustic transfer around 1.1 kHz to 1.8 kHz.^2^^,^^8^^,^^11^^,^^14^^,^^22^

Although the maturation of the OE and ME is known to be responsible for modifying aspects relevant to the conduction of sound energy, the relationships between the measurements obtained in the WAI and the differences between age groups throughout childhood are not fully characterized. This research sought to investigate essential properties for acoustic transmission, such as admittance, absorbance, and resonance frequency, considering the influence of anatomical and functional changes on the development of the auditory system, from birth to 11 years of age.

## Materials and methods

This prospective cross-sectional observational study was conducted in accordance with the STROBE (Strengthening the Reporting of Observational Studies in Epidemiology) guidelines. The STROBE checklist was used as a reference to ensure the quality and clarity of the study’s methodology, results, and interpretations. This study is part of one of the stages of a project approved by the institution's Research Ethics Committee. The Informed Consent Form was signed by the legal guardians of all participants.

The recruitment and data collection of participants took place in a maternity hospital and in an elementary school in São Paulo, Brazil, between October 2021 and July 2024. After voluntary parental/guardian consent was obtained, the participants were evaluated at the facilities of *Irmandade da Santa Casa de Misericórdia de São Paulo*, in an acoustically treated room. The inclusion criteria were: full-term birth, adequate prenatal and perinatal characteristics (including weight, height, head circumference, and Apgar score), absence of risk indicators for hearing loss,^23^ and complaints related to hearing or neuropsychomotor development. The data were obtained from reading medical records and anamneses with the parent/guardian. Hearing normality was ensured by specific procedures for each age group,^23^, ^24^, ^25^, ^26^, ^27^ presented in Table 1. Both participants' ears were tested when possible. The first ear tested was the one that was most easily positioned. The exclusion criteria were non-compliance with the complete testing protocol or children who were unable to cooperate with the assessment.

**Table 1:** Description of participants inclusion criteria procedures by age group.

Age Groups	Procedures^a^	Equipment^b^	Acoustic Stimuli	Standard Criteria for Inclusion
**NB** **6‒8m**	Automated Auditory Brainstem Response (AABR)	Titan (Interacoustic) ABRIS 440 module ER 3A insert earphones Surface electrodes	CE-Chirp stimuli at 35 dB HL^22^^,^^23^	“Pass” (screening) results according to equipment parameters
	Tympanometry	Titan (interacoustics)	1k Hz probe tone at 85 dB SPL Downsweep (+200 daPa to −400 daPa)^22^^,^^24^	Positive peak tympanogram PPT between +150 daPa and −200 daPA
	Ipsilateral acoustic reflexes	IMP440 module	Using 226 Hz probe tone, automatic presentation of 750 ms continuous tones on PPT, ascending method ‒starting at 80 dB HL^22^^,^^24^	Responses at all frequencies (0.5k to 4k Hz) Minimum amplitude: 0.2mL^23^
**3‒5y**	Pure Tone Audiometry (PTA)	AC33 audiometer (Interacoustics)	Warble tones modulated from 0.25k to 8k Hz in each ear^23^^,^^24^	Air conduction thresholds up to 15 dB HL in both ears
	Speech Recognition Threshold (SRT)	Supro-aural headphones TDH-39 (Telephonics) Double-wall silent acoustic booth	Trisyllabic words in descending-ascending technique^23^^,^^24^	
**6‒8y**	Tympanometry	Titan (Interacoustics) IMP440 module	226 Hz probe tone at 85 dB SPL Downsweep (+200 daPa to −400 daPa)^22^^,^^24^	“Type A” tympanograms PPT between +100 daPa and −100 daPa
**9‒11y**	Ipsilateral acoustic reflexes		Using 226 Hz probe tone, automatic presentation of 750 ms continuous tones on PPT, ascending method-starting at 80 dB HL^22^^,^^24^	Responses at all frequencies (0.5k to 4k Hz) Minimum amplitude: 0.2mL
**All**	Transient Otoacoustic Emissions (TEOAE)	Titan (interacoustics) TEOAE 440 module	Non-linear broadband clicks at 83 dB SPL equivalent peak	Response (signal) ≥−10 dB SPL ‒ at least 3 frequency ranges
**NB to 9‒11y**	Distortion Product Otoacoustic Emissions (DPOAE)	Titan (Interacoustics) DPOAE 440 module	Minimum 260 sweeps accepted. Results recorded for frequency ranges from 1k to 5k Hz^25^	Signal-to-noise ratio ≥6 dB SPL ‒ at least 4 frequency ranges Reproducibility ≥90 %
			Primary tone pairs *f*_1_ and *f*_2_ at 65 dB and 55 dB NPS, respectively *f*_2_ ranging from 0.5k Hz to 10k Hz *f*_2_/*f*_1_ ratio fixed at 1.22^25^	Response (signal) ≥−10 dB SPL ‒ at least 8/12 frequencies
				Signal-to-noise ratio ≥6 dB NPS ‒ at least 8/12 frequencies

a All procedures were carried out on the same day as the WAI data collection.

b All equipment was properly checked and calibrated in accordance with current standards.

The sample consisted of 117 participants between six days and 11 years, 10 months of age. Five groups were established depending on age range: 35-Newborns (NB), 15 infants aged six to eight months (6‒8 m), 10 children aged three to five years (3‒5y), 28 aged six to eight years (6‒8y), and 29 children aged nine to eleven (9‒11y). In the sample calculation (ANOVA – for ECV), a minimum number of five participants per group was defined with a significant level of 5 % (alpha = 0.05) and test power of 80 % (β = 0.2). As this study is part of a larger study that included other measures, there was a larger number of participants in each group.

In order to ensure quality control of the recordings and minimize background noise, an attempt was made to adjust the probe with deep insertion into the EAC, without the need to hold it. The tests started after satisfactory conditions were found in the biological calibration with appropriate sealing with a silicone ear tip. Normal results in Transient Otoacoustic Emissions (TEOAE) and distortion product (DPOAE)^24^ after tympanometry with the same equipment increased confidence about adequate sealing and maintenance of probe positioning. The eligibility of the participants and the careful execution of the research procedures sought to minimize potential confounding factors.

The WBT440 module in the Titan handheld tympanometer (Interacoustics*®*) was used. Installation, annual (ISO 389–1; IEC 60,645 standards) and daily (in test cavity) calibration was carried out in accordance with the manufacturer's technical specifications. WAI data was recorded and analyzed using OtoAccess version 3.0 (Interacoustics®), installed on a Windows-based portable computer (HP brand), coupled to the device hardware.

The broadband probe array delivered approximately 32 clicks (from 0.2k to 8k Hz), at a presentation rate of 21.5/s, at 96 dB SPL peSPL. The pressure sweep was downward from 200 to −400 daPa, with a speed of 300 daPa/s. The measurements analyzed were: ear canal volume ECV – obtained with probe tone at 226 Hz, acoustic admittance compensated with probe tone at 1k Hz (Y_TM_1kHz) and 226 Hz (Y_TM_226Hz), Tympanometry Peak Pressure (TPP), Tympanometric Width (TW), acoustic absorbance, according to frequency, obtained at atmospheric pressure (close to 0 daPa) and RF. To optimize the number of absorbance analysis points, indices were extracted at the 16 frequencies closest to the midpoints every 1/3 of an octave: 0.25k, 0.3k, 0.4k, 0.5k, 0.6k, 0.8k, 1k, 1.25k, 1.5k, 2k, 2.5k, 3k, 4k, 5k, 6k, and 8 kHz.^4^^,^^25^^,^^27^^,^^28^

The correlations between WAI measures were also analyzed, such as: ECV and acoustic absorbance; ECV and RF; Y_TM_ 1 kHz or Y_TM_ 226 Hz and absorbance; Y_TM_ 1 kHz or Y_TM_ 226 Hz and RF; and absorbance and RF.

Statistical analyses were done using SPSS version 25. The Shapiro-Wilk normality test showed an asymmetrical distribution in relation to age groups in the results of this research. Thus, for inferential analysis, the effect of age and its influence on the WAI measurements were analyzed using the Kruskal-Wallis test. When a statistically significant difference was observed, multiple comparisons between age groups were performed between groups using the Mann-Whitney test. Comparison of results between sexes and ears in each group was performed using the Wilcoxon test. The Pearson correlation coefficient (r) was used for linear correlation analyses. To analyze the influence of age on acoustic absorbance measurements, a simple linear regression model (ANOVA) was also used. A significant level of *p* < 0.05 was considered.

## Results

A total of 118 individuals were recruited, and one infant in the 6‒8 m group was not included in the study due to tympanometry suggestive of conductive alteration and absence of acoustic reflexes in both ears. Among 117 participants, the ears excluded were those with recordings of otoacoustic emissions with inadequate signal-to-noise ratio,^24^ non-cooperation with the tests or crying during one of the procedures (eight ears in the NB group and three ears in 6‒8 m), or excess cerumen (two ears in group 6‒8y and one in group 9‒11y). Thus, WAI measurements were analyzed in 62 ears in the NB group, 27 in 6‒8 m, and 20, 54, and 57 ears in the 3‒5y, 6‒8y, and 9‒11y groups, respectively, totaling 220 ears.

There was similarity between the number of right (112) and left (108) ears examined (Chi-Square test = 0.0727) and symmetric distribution of female (58) and male (59) individuals in the entire sample (Chi-Square test = 0.0727) square = 0.106). The Newborns (NB) were born at term – an average of 39.2 weeks of gestational age and were between six and 30 days old at the time of evaluation (average 16 + 6 days).

In all WAI measurements analyzed, the Wilcoxon test showed no difference depending on the ear or gender, both in the total sample and in the individual groups. For this reason, the results obtained by ear and sex were grouped together for each age group.

### Ear canal volume (ECV), tympanometry peak pressure (TPP), tympanometric width (TW), and acoustic admittance (Y_TM_)

**Fig. 1:**
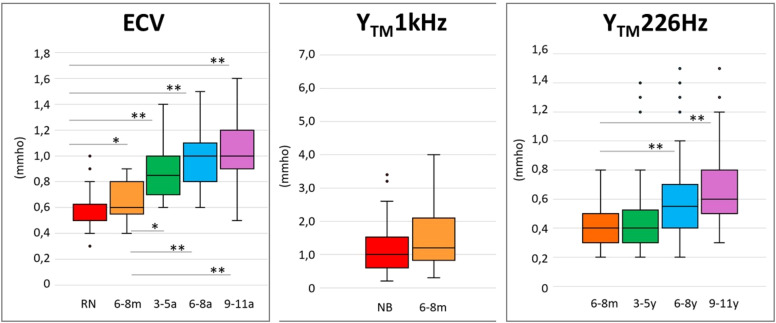
Boxplots by age group of the distribution of the Ear Canal Volume (ECV) obtained with probe tone at 226 Hz, compensated acoustic admittance (at the height of the tympanic membrane) with probe tone at 1k Hz (Y_TM_ 1 kHz) and 226 Hz (Y_TM_ 226 Hz). Medians, first and third quartiles, minimum and maximum values are observed in each age group. NB, Newborn; 6‒8 m, Infants from 6 to 8 months; 3‒5y, Children aged 3 to 5; 6‒8y, Children aged 6 to 8; 9‒11y, Children aged 9 to 11; ECV, Ear Canal Volume, obtained from tympanometry with a 226 Hz probe; Y_TM_ 1k Hz, Acoustic admittance compensated with tone probe at 1k Hz; Y_TM_ 226 Hz, Acoustic admittance compensated with probe tone at 226 Hz; mmho, Milimho. The dots indicate outliers. Bars indicate statistical significance for the age effect by Mann-Whitney test *p ≤ 0.05 and **p ≤ 0.01.

The ECV, TPP, TW, Y_TM_1kHz and Y_TM_226Hz measurements obtained in the entire sample were compatible with normal standards expected^25^ in each age group, and the results are presented in Table 2. The results of the Kruskal-Wallis analysis (p) for the effect of age in each measurement are described in the same table. Fig. 1 illustrates the distribution of ECV, Y_TM_ 1 kHz, and Y_TM_ 226 Hz measurements in each age group.

**Table 2:** Descriptive measurements of ECV, TPP, TW, Y_TM_1kHz and Y_TM_226Hz by age group and statistical comparison by the non-parametric Kruskal-Wallis test and multiple comparisons based on age group by the Mann-Whitney test.

							Age effect											
		Group					Kruskal-Wallis test	Mann-Whitney test (age groups compared)										
								NB × 6–8m	NB × 4–5y	NB × 6–8y	NB × 9–11y	6–8 *m* × 3–5y	6–8 *m* × 6–8y	6–8 *m* × 9–11y		3–5y × 6–8y	3–5y × 9–11y	6–6y × 9–11y
		NB	6–8m	3–5y	6–8y	9–11y	p-value	p-value										
**ECV (mmho)**	**Median**	0.5	0.6	0.9	1.0	1.0	**0.000**	**0.028**	**0.000**	**0.000**	**0.000**	**0.014**	**0.000**	**0.000**	0.195		0.053	0.219
	**Mean (SD)**	0.55 (±0.13)	0.65 (±0.16)	0.9 (±0.22)	1.0 (±0.23)	1.05 (±0.25)												
**TPP (daPa)**	**Median**	−18	−3	−33	−2	−4	0.080	0.138	0.269	0.135	0.158	0.053	0.573	0.454	0.059		0.051	0.719
	**Mean (SD)**	−23 (±37)	−6 (±32)	−44 (±60)	−7 (±23)	−9 (±22)												
**TW (daPa)**	**Median**	113	104	121	112	108	0.068	0.694	0.386	0.816	0.261	0.276	0.011	0.526	0.411		0.069	0.066
	**Mean (SD)**	109 (±32)	108 (±27)	135 (±55)	111 (±24)	107 (±29)												
**Y_TM_ 1KHz (mmho)**	**Median**	0.9	1.2				0.233	0.253										
	**Mean (SD)**	1.2 (±0.72)	1.55 (±1.10)															
**Y_TM_ 226Hz (mmho)**	**Median**	0.4	0.4	0.6	0.6	0.4	**0.005**					0.510	**0.006**	**0.005**	0.135		0.099	0.494
	**Mean (SD)**	0.4 (±0.17)	0.5 (±0.37)	0.6 (±0.29)	0.6 (±0.28)	0.4 (±0.17)												

ECV, Ear Canal Volume; mmho, millimho; TPP, Tympanometry Peak Pressure; daPa, Decapascal; TW, Tympanometric Width; SD, Standard Deviation; k Hz, Kilohertz; Y_TM_ 1 kHz, Acoustic admittance with 1k Hz probe tone; Y_TM_ 226 Hz, Acoustic admittance with 226 Hz probe tone; SD, Standard Deviation; NB, Newborns; 6‒8 m, Infants from 6 to 8 months; 3‒5y, Children from 3 to 5 years old; 6‒8y, Children from 6 to 8 years old; 9‒11y, Children from 9 to 11 years old.

Multiple comparisons (Mann-Whitney test) for ECV showed a significant difference (*p* < 0.03) between the results of NB and other groups. When comparing 6‒8 m with the other age groups, there was also significance (*p* < 0.03) (Fig. 1). ECV measurements showed no significant difference in multiple comparisons between children aged three to eleven years (3‒5y, 6‒8y, and 9‒11y) (Table 2).

For TPP and TW measurements obtained in all groups, results were found to be normal^25^ (Fig. 1), and there was no age effect (Kruskal-Wallis – *p* > 0.05 in all analyses) (Table 2).

The acoustic admittance results (Y_TM_) were compatible with normal middle ear functions^25^ in all age groups (Fig. 1). There was no statistically significant difference in the comparison of Y_TM_1kHz between NB and 6‒8 m (Mann-Whitney – *p* = 0.233). For Y_TM_ 226 Hz, the results showed no difference in the multiple comparison (Mann-Whitney) between 6‒8 m and 3‒5y groups, nor in the comparison between 3‒5y, 6‒8y and 9‒11y groups, but the statistical difference was evident in the comparison between 6‒8 m and the older age groups (6‒8y and 9‒11y) (*p* < 0.02) (Table 2).

### Acoustic absorbance

**Fig. 2:**
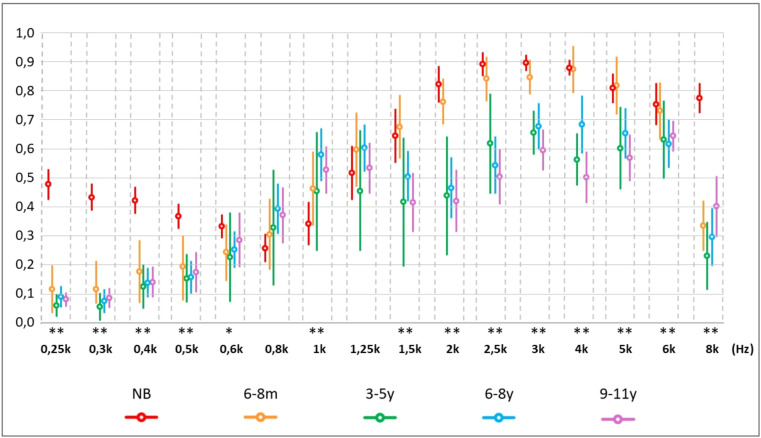
Distribution of absorbance measurements by frequency in each age group with median and range of variation from 5 % to 95 %. NB, Newborn; 6‒8 m, Infants from 6 to 8 months; 3‒5y, Children aged 3 to 5; 6‒8y, Children aged 6 to 8; 9‒11y, Children aged 9 to 11; k Hz, kiloHertz. Asterisks (*) indicate the frequencies in which statistical significance was found for the age effect by the Kruskal-Wallis test * *p* ≤ 0.05 and ** *p* ≤ 0.01.

The Kruskal-Wallis test showed an effect of age on the absorbance measurement (*p* < 0.03) for all frequencies, except 0.8k Hz and 1.25k Hz (Fig. 2). In the ANOVA model, there was a significant effect of age (*p* < 0.02) in the frequency bands 0.25k–0.5k Hz, 0.8k–1k Hz, and 1.5k–8k Hz.

Absorbance from 0.25k to 0.5k Hz and at 8k Hz was higher in the NB group than in all other age groups (Mann-Whitney – *p* < 0.04 for all comparisons). The average indices at low frequencies in the NB group were greater than 0.4, while those found from six months onwards did not reach 0.2 (Table 3).

**Table 3:** Descriptive absorbance measurements by frequency in each age group.

	Group									
	NB		6‒8m		3‒5y		6‒8y		9‒11y	
	Median & CI 5 %‒95 %	Mean & SD	Median & CI 5 %‒95 %	Mean & SD	Median & CI 5 %‒95 %	Mean & SD	Median & CI 5 %‒95 %	Mean & SD	Median & CI 5 %‒95 %	Mean & SD
**0.25k**	0.49	0.48	0.08	0.12	0.05	0.06	0.06	0.09	0.05	0.08
	0.43‒0.53	(±0.14)	0.04‒0.20	(±0.14)	0.02‒0.10	(±0.06)	(0.05‒0.13)	(±0.09)	0.06‒0.11	(±0.07)
**0.3k**	0.43	0.44	0.07	0.12	0.03	0.06	0.03	0.07	0.06	0.09
	0.39–0.48	(±0.13)	0.03‒0.21	(±0.16)	0.01‒0.10	(±0.07)	0.03‒0.11	(±0.10)	0.05‒0.12	(±0.09)
**0.4k**	0.41	0.42	0.14	0.18	0.09	0.13	0.10	0.14	0.09	0.14
	0.38‒0.47	(±0.13)	0.07‒0.29	(±0.18)	0.05‒0.20	(±0.10)	0.09‒0.19	(±0.11)		0.09‒0.19
**0.5k**	0.37	0.36	0.17	0.20	0.11	0.15	0.12	0.16	0.11	0.18
	0.32‒0.41	(±0.12)	0.09‒0.30	(±0.18)	0.07‒0.24	(±0.12)	0.10‒0.21	(±0.14)	0.11‒0.24	(±0.18)
**0.6k**	0.35	0.33	0.24	0.16	0.23	0.23	0.25	0.20	0.29	0.35
	0.29‒0.37	(±0.11)		0.15‒0.34	(±0.17)	0.07‒0.38	(±0.21)	0.19‒0.31	(±0.16)	0.19‒0.38
**0.8k**	0.25	0.25	0.30	0.31	0.30	0.33	0.36	0.39	0.29	0.37
	0.21‒0.30	(±0.13)	0.19‒0.43	(±0.20)	0.13‒0.53	(±0.27)	0.30‒0.48	(±0.22)	0.27‒0.47	(±0.25)
**1k**	0.32	0.34	0.49	0.46	0.40	0.45	0.58	0.58	0.55	0.53
	0.27‒0.41	(±0.20)	0.34‒0.59	(±0.21)	0.25‒0.66	(±0.27)	0.49‒0.67	(±0.23)	0.45‒0.61	(±0.21)
**1.25k**	0.53	0.52	0.60	0.60	0.35	0.46	0.57	0.60	0.61	0.53
	0.43‒0.61	(±0.26)	0.47‒0.73	(±0.21)	0.25‒0.66	(±0.28)	0.52‒0.68	(±0.20)	0.45‒0.62	(±0.23)
**1.5k**	0.67	0.64	0.76	0.68	0.46	0.42	0.51	0.50	0.42	0.42
	0.55‒0.73	(±0.26)	0.57‒0.79	(±0.18)	0.20‒0.64	(±0.30)	0.42‒0.59	(±0.22)	0.31‒0.52	(±0.26)
**2k**	0.88	0.82	0.78	0.77	0.48	0.44	0.52	0.46	0.42	0.42
	0.76‒0.88	(±0.17)	0.69‒0.84	(±0.13)	0.23‒0.64	(±0.28)	0.36‒0.57	(±0.26)	0.31‒0.53	(±0.28)
**2.5k**	0.93	0.88	0.89	0.84	0.62	0.62	0.57	0.54	0.51	0.50
	0.84‒0.93	(±0.13)	0.77‒0.92	(±0.13)	0.45‒0.79	(±0.23)	0.44‒0.64	(±0.25)	0.41‒0.60	(±0.24)
**3k**	0.90	0.89	0.88	0.85	0.66	0.66	0.72	0.68	0.58	0.60
	0.85‒0.92	(±0.10)	0.79‒0.91	(±0.09)	0.58‒0.73	(±0.11)		0.60‒0.76	(±0.20)	0.53‒0.67
**4k**	0.89	0.87	0.95	0.88	0.55	0.56	0.74	0.68	0.55	0.50
	0.84‒0.90	(±0.08)	0.79‒0.96	(±0.14)	0.47‒0.65	(±0.13)	0.58‒0.78	(±0.25)	0.41‒0.59	(±0.23)
**5k**	0.84	0.80	0.86	0.82	0.60	0.60	0.69	0.65	0.55	0.57
	0.75‒0.85	(±0.15)	0.72‒0.92	(±0.16)	0.46‒0.74	(±0.19)	0.56‒0.74	(±0.22)	0.49‒0.65	(±0.21)
**6k**	0.82	0.75	0.79	0.73	0.69	0.63	0.66	0.62	0.64	0.64
	0.67‒0.82	(±0.21)	0.63‒0.83	(±0.16)	0.50‒0.77	(±0.18)	0.53‒0.70	(±0.21)	0.59‒0.70	(±0.14)
**8k**	0.79	0.77	0.35	0.34	0.18	0.23	0.23	0.29	0.33	0.40
	0.72‒0.82	(±0.14)	0.25‒0.42	(±0.15)	0.11‒0.35	(±0.17)	0.19‒0.39	(±0.25)	0.30‒0.51	(±0.27)

k Hz, Kilohertz; CI, Confidence Interval; SD, Standard Deviation; NB, Newborns; 6‒8 m, Infants from 6 to 8 months; 3‒5y, Children from 3 to 5 years old; 6‒8y, Children from 6 to 8 years old; 9‒11y, Children from 9 to 11 years old.

At frequencies from 2k Hz to 6k Hz, the average indices were similar in NB and 6‒8 m, which were higher than the values in the other groups (3‒5y, 6‒8y and 9‒11y) (Table 3) and statistically significant difference was found in multiple comparisons between NB and the groups 3‒5y, 6‒8y and 9‒11y, as well as in comparisons of 6‒8 m with age groups from three to eleven years old (3‒5y, 6‒8y and 9‒11y) (Mann-Whitney – *p* < 0.05 in all comparisons) (Table 4).

**Table 4:** Comparison of absorbance values as a function of age group by the non-parametric Kruskal-Wallis test and multiple comparisons by the Mann-Whitney test.

Kruskal-Wallis test				Mann-Whitney test									
				Age groups compared									
Frequency (Hz)	Groups	NB		NB × 6–8m	NB × 3–5y	NB × 6–8y	NB × 9–11y	6–8 *m* × 3–5y	6–8 *m* × 6–8y	6–8 *m* × 9–11y	3–5y × 6–8y	3–5y × 9–11y	6–8y × 9–11y
		6–8m											
		3–5y											
		6–8y											
		9–11y											
**0.226k**	p-value	**0.000**	p-value	**0.000**	**0.000**	**0.000**	**0.000**	0.277	0.738	0.061	0.426	0.936	0.162
**0.25k**		**0.000**		**0.000**	**0.000**	**0.000**	**0.000**	0.120	0.267	0.056	0.618	0.822	0.362
**0.3k**		**0.000**		**0.000**	**0.000**	**0.000**	**0.000**	0.264	0.222	0.946	0.934	0.246	0.160
**0.4k**		**0.000**		**0.000**	**0.000**	**0.000**	**0.000**	0.538	0.739	0.508	0.765	0.962	0.492
**0.5k**		**0.000**		**0.000**	**0.000**	**0.000**	**0.000**	0.446	0.371	0.271	0.934	0.595	0.762
**0.6k**		**0.021**		**0.017**	**0.035**	**0.013**	**0.008**	0.482	0.894	0.697	0.476	0.552	0.719
**0.8k**		0.218		0.275	0.554	**0.036**	**0.044**	0.815	0.423	0.678	0.540	0.479	0.798
**1k**		**0.002**		**0.033**	0.197	**0.000**	**0.001**	0.770	0.235	0.407	0.208	0.385	0.513
**1.25k**		0.419		0.135	0.808	0.122	0.280	0.242	0.852	0.517	0.296	0.499	0.719
**1.5k**		0.007		0.556	0.274	0.093	**0.015**	0.061	**0.001**	**0.002**	0.868	0.563	0.444
**2k**		0.000		0.540	**0.001**	**0.000**	**0.000**	**0.006**	**0.000**	**0.000**	0.791	0.499	0.678
**2.5k**		0.000		0.162	**0.004**	**0.000**	**0.000**	**0.030**	**0.000**	**0.000**	0.289	0.222	0.737
**3k**		0.000		0.141	**0.000**	**0.000**	**0.000**	**0.001**	**0.009**	**0.000**	0.921	0.143	0.138
**4k**		0.000		0.117	**0.000**	**0.000**	**0.000**	**0.000**	**0.001**	**0.000**	0.068	0.607	**0.009**
**5k**		0.000		0.281	**0.001**	**0.002**	**0.000**	**0.002**	**0.004**	**0.000**	0.398	0.797	0.196
**6k**		0.001		0.418	**0.039**	**0.002**	**0.003**	**0.040**	**0.003**	**0.007**	0.551	0.872	0.655
**8k**		0.000		0.000	**0.000**	**0.000**	**0.000**	**0.002**	0.056	0.517	0.417	**0.038**	0.064

k Hz, Kilohertz; NB, Newborns; 6‒8 m, Infants from 6 to 8 months; 3‒5y, Children from 3 to 5 years old; 6‒8y, Children from 6 to 8 years old; 9‒11y, Children from 9 to 11 years old.

In the NB group, the highest absorbance levels were concentrated in the range from 1.25k to 8k Hz. Likewise, in 6‒8 m, the highest absorbance levels were obtained from 1.25k Hz, however, up to the frequency 6k Hz, with a significant reduction observed at 8k Hz. Low absorbance levels at 8k Hz were also found in the other groups (Fig. 2). At frequencies of 0.8k and 1k Hz, the lowest average absorbance indices of the entire sample were obtained in the NB group, with a gradual increase from 6‒8 m, however, with no statistical difference in relation to the other groups. At 1k Hz, the means were similar in the 6‒8 m and 3‒5y groups, being higher than in NB and lower than in 6‒8y and 9‒11y. The levels were gradually higher with increasing age, but similar between 6‒8y and 9‒11y. In the 3‒5y group, higher measurements were obtained in the range of 1.5k to 6k Hz, and in the 6‒8y and 9‒11y groups, the highest rates occurred from 1k to 6k Hz (Table 3).

### Resonance frequency (RF)

**Fig. 3:**
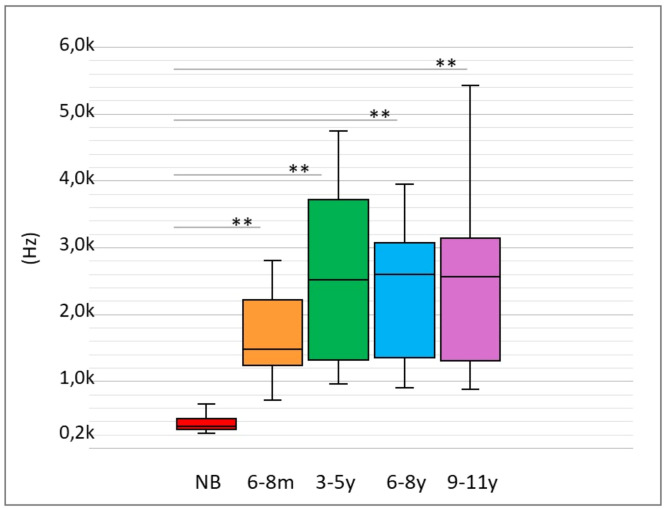
Boxplots of resonance frequency distribution by age group. Medians, first and third quartiles, minimum and maximum values are observed in each age group. Caption: NB, Newborn; 6‒8 m, Infants from 6 to 8 months; 3‒5y, Children aged 3 to 5; 6‒8y, Children aged 6 to 8; 9‒11y, Children aged 9 to 11; Hz, Hertz. Upper bars indicate statistical significance for the age effect by Mann-Whitney test; ** *p* ≤ 0.01.

RF data showed a clear increase with age. Especially in NB, RF was lower than in infants (6‒8 m), and there were no apparent changes from the age group 3‒5y to 9‒11y. There was great variability in RF in all age groups, except for NB (Fig. 3).

The effect of age on RF was evident (Kruskal-Wallis – *p* = 0.000), and there was a significant difference when comparing NB and the other age groups (Mann-Whitney – *p* = 0.000 for all comparisons). However, although greater RF was observed in the 3‒5y, 6‒8y, and 9β11y groups than in the 6‒8 m group, there was no statistically significant difference in multiple comparisons between these groups (Table 5).

**Table 5:** Descriptive measurements of resonance frequency by age group and statistical comparison by the non-parametric *Kruskal-Wallis* test and multiple comparisons based on age group by the Mann-Whitney test.

		Descriptive measurements				Age effect				
		Median (kHz)	CI (k Hz)		Mean (SD) (kHz)	Kruskal-Wallis test	Mann-Whitney test			
			5 %	95 %						
						p-value	p-value (Age groups compared)			
**Group**	**NB**	0.321	0.318	0.38	0.353 (±0.097)	**0.000**	NB × 6–8m			
							**0.000**			
	**6–8m**	1.479	1.295	1.997	1.649 (±0.595)		NB × 3–5y	6–8 *m* × 3–5y		
	**3–5y**	2.514	1.565	3.506	2.586 (±1.282)		**0.000**	0.108		
	**6–8y**	2.599	1.944	2.659	2.299 (±0.903)		NB × 6–8y	6–8 *m* × 6–8y	3–5y × 6-By	
							**0.000**	0.052	0.501	
	**9–11y**	2.567	1.919	2.793	2.354 (±1.136)		NB × 9–11y	6–8 *m* × 9–11y	3–5y × 9–11y	6–8y × 9–11y
							**0.000**	0.076	0.540	0.845

CI, Confidence Interval; SD, Standard Deviation; k Hz, Kilohertz; NB, Newborns; 6‒8 m, Infants from 6 to 8 months; 3‒5y, Children from 3 to 5 years old; 6‒8y, Children from 6 to 8 years old; 9‒11y, Children from 9 to 11 years old.

### Correlations between wideband acoustic immittance (WAI) measures

In the total sample, there was a significant negative correlation between ECV and absorbance indices at 0.25k and 0.3k Hz and from 2k to 4k Hz (Pearson correlation coefficients – *r* ≥ 0.270 and *p* < 0.01).

In the total sample, there was a significant correlation between ECV and absorbance indices, with negative Pearson correlation coefficients (*r*), being higher at 0.25k, 0.3k Hz, and 2k–4k Hz (*p* < 0.01). That is, in ears with smaller EAC volumes, higher absorbance levels were obtained at most frequencies (Table 6). ECV also showed a positive correlation with RF in the total sample (*r* ≥ 0.390, *p* < 0.01), but there was no significant correlation between RF and Y_TM_ 1 kHz, nor between RF and Y_TM_ 226 Hz (except negative correlation in 9‒11y – *p* = 0.046).

**Table 6:** Pearson correlation analyses for Ear Canal Volume (ECV), acoustic admittance at 1k Hz (Y_TM_1kHz) and 226 Hz (Y_TM_226Hz), absorbance, and Resonance Frequency (RF) measures in the total sample.

		ECV		Y_TM_ 1kHz		Y_TM_ 226Hz		RF	
		Total sample	Groups with significant *r*	Total sample	Groups with significant *r*	Total sample	Groups with significant *r*	Total sample	Groups with significant *r*
**Y_TM_ 1kHz**	**Correlation type**	**Positive**	NB^a^ 6–8m^a^						
	**P. coefficient (*r*)**	**>0.410**							
	**p-value**	**<0.03**^a^							
**Y_TM_ 226kHz**	**Correlation type**	**Positive**	6–8m^b^ 3–5y^b^ 6–8y						
	**P. coefficient (*r*)**	**>0.400**							
	**p-value**	**<0.03**^a^							
**0.25k‒0.6kHz**	**Correlation type**	**Negative**	No correlation found by group	No correlation	No correlation found by group	**Positive** **>0.320** **<0.05**^a^	3–5y^a^ 6–8y^b^ 9–11y^b^	**Negative** **>0.570** **<0.01**^b^	9–11y^a^
	**P. coefficient (*r*)**	**>0.310**							
	**p-value**	**<0.03**^a^							
**0.8k‒1k Hz**	**Correlation type**	**Positive**	No correlation found by group	**Positive** **>0.400** **<0.02**^a^	No correlation found by group	**Positive** **>0.505** **<0.01**^b^	3–5y^a^ 6–8y^b^ 9–11y^b^	No correlation	9–11y^a^ (negative)
	**P. coefficient (*r*)**	**>0.440**							
	**p-value**	**<0.01**^b^							
**1.5k‒6k Hz**	**Correlation type**	**Negative**	No correlation found by group	No correlation	No correlation found by group	No correlation	No correlation found by group	**Negative** **>0.380** **<0.05**^a^	No correlation found by group
	**P. coefficient (*r*)**	**>0.440**							
	**p-value**	**<0.01**^b^							
**8kHz**	**Correlation type**	**Negative**	No correlation found by group	**Negative** **>0.300** **<0.04**^a^	No correlation found by group	No correlation	No correlation found by group	**Negative** **>0.410** **<0.01**^b^	No correlation found by group
	**P. coefficient (*r*)**	**>0.410**							
	**p-value**	**<0.01**^b^							
**RF**	**Correlation type**	**Positive**	No correlation found by group	No correlation	No correlation found by group	No correlation	9–11^a^ (negative)		
	**P. coefficient (*r*)**	**>0.390**							
	**p-value**	**<0.01**^b^							

ECV, Ear Canal Volume; Y_TM_ 1 kHz, Acoustic admittance with 1k Hz probe tone; Y_TM_ 226 Hz, Acoustic admittance with 226 Hz probe tone; RF, Resonance Frequency; k Hz, kilohertz; P. coefficient (*r*), Pearson correlation coefficient; NB, Newborns; 6‒8 m, Infants from 6 to 8 months; 3‒5y, Children from 3 to 5 years old; 6‒8y, Children from 6 to 8 years old; 9‒11y, Children from 9 to 11 years old.

Asterisks (*) indicate the frequencies in which statistical significance was found for the age effect by the Kruskal-Wallis test ^a^*p* ≤ 0.05 and ^b^*p* ≤ 0.01.

Absorbance indices at 0.25k Hz showed a significant positive correlation with Y_TM_ 226 Hz values among children aged three to eleven years old (*r* ≥ 0.380 and *p* < 0.05). This correlation was not found between the 1k Hz absorbance and Y_TM_ 1 kHz in NB and 6‒8 m groups, but occurred in the total sample (*r* = 0.485 and *p* < 0.01). The highest absorbance levels in the 0.8k–1.25k Hz frequency range were also accompanied by a significant increase in Y_TM_ 226 Hz in the entire sample from six months of age onwards (*r* ≥ 0.210 and *p* < 0.03).

The highest absorbance at frequencies from 0.25k to 0.4k kHz Hz in the entire sample was found in the NB group, coinciding with the RF obtained. In all groups, the RF coincided with absorbance indices ≥0.4; however, the highest average absorbance was observed at higher frequencies. In NB, for example, average levels from 2k to 8k Hz exceeded 0.75. In 6‒8 m group, there was high absorbance (average 0.68) at a frequency of 1.5k Hz, close to the average RF for this age group, but levels were higher between 2k and 6k Hz. From three years of age onwards (3‒5y, 6‒8y and 9‒11y), RF was verified at around 2.5k Hz, and the highest absorbance averages (around 0.5 to 0.6) were obtained from 2.5k to 6k Hz.

Table 6 summarizes the results of Pearson correlation, indicating the type, coefficient (*r*) and level of significance (p) between the variables. In most analyses, there was statistical significance in the total sample, but generally, they were not significant when stratified by age group.

## Discussion

This study carefully analyzed WAI measurements across a wide age range, allowing the observation of a series of changes related to auditory development at different stages. All participants showed normal results in tests frequently used across all ages, which are considered to have high sensitivity and specificity in other studies.^4^^,^^6^^,^^8^^,^^11^, ^12^, ^13^, ^14^, ^15^^,^^18^^,^^29^^,^^30^

Once conductive alterations were ruled out in all participants, to facilitate the appreciation of the acoustic absorbance results of this study in comparison to others, only reports of measurements obtained at ambient pressure or close to 0 daPa will be mentioned, in individuals who presented normal results in audiological tests, following criteria similar to the current research. Although increasing the precision in identifying ME disorders, pressurization in the external auditory canal does not seem to influence absorbance measurements in healthy ears.^8^^,^^30^, ^31^, ^32^, ^33^ The accuracy of investigation at ambient pressure was demonstrated across different ages^9^ and, especially in infants, would reduce the interference of changes in the shape and volume of the external auditory canal during the procedure.^28^

### Ear canal volume (ECV)

In agreement with other investigations, no significant differences were found regarding sex and ear (right vs. left) in the ECV,^31^ TPP, and TW,^17^^,^^22^^,^^32^ acoustic admittance with both probe tones – Y_TM_ 1 kHz and Y_TM_ 226 Hz,^15^^,^^17^ absorbance or reflectance by frequency,^14^, ^15^, ^16^^,^^19^^,^^33^^,^^34^ and RF.^12^^,^^14^^,^^20^ Differences related to ear or sex in some of these measures are generally of low magnitude.^17^^,^^18^^,^^21^ Inconsistencies between studies may be influenced by different sample sizes, age, height, weight, and ethnic factors of the participants, procedures used, or other unknown aspects. However, as these differences are subtle, they would have no clinical relevance nor justify differentiated normative criteria.^8^^,^^19^^,^^21^

The ECV results of this research were consistent with previous experiments with newborns, infants,^6^^,^^8^^,^^22^^,^^27^ and children.^17^^,^^18^ The effect of age on this measure aligns with publications that reported a systematic increase with growth and supports the claim that this is an acoustically relevant factor.^8^^,^^17^^,^^18^ The statistically significant difference in the comparison of this measure between NB and 6‒8 m, as well as in the comparison of both groups with the other age ranges, showed that the most important structural changes in the outer ear occur mainly during the first year of life, as previously described.^22^ The increase in mean ECV, but with less pronounced differences from the age of three onwards, shows that changes are more gradual after this age, and measures similar to those of adults are expected to occur only after the age of 12.^2^^,^^17^^,^^18^^,^^20^

There were some differences in the average ECV of the NB and 6‒8 m groups compared to those described in the experiment by Alaerts and colleagues,^22^ which could be related to grouping by age since the study did not differentiate between infants up to three months old and grouped infants from three to nine months. A slight increase in ECV between nine and 32 months was also reported. Therefore, the statistical difference found in the comparison between the mean ECV of the 6‒8 m and 3‒5y groups might indicate a significant increase in the size of the ECV even after three years of age, as mentioned in other publications.^2^^,^^20^

### Tympanometry peak pressure (TPP), tympanometric width (TW), and acoustic admittance (Y_TM_)

The ears examined showed TPP results similar to other studies conducted with children without ME alterations.^11^^,^^17^^,^^18^^,^^22^^,^^30^ The use of a descending pressure sweep has been recommended^4^ and was justified by its advantage in confirming a hermetic seal, minimizing the effects of ECV collapse, and allowing reliable admittance and absorbance data collection within the studied age range. However, predominantly negative mean TPP values, still within normal criteria, are more common in descending sweeps at all ages.^4^

The TW results are consistent with previous studies conducted on children with normal hearing,^17^^,^^22^^,^^32^ where TW is treated as supplementary in determining the state of the middle ear.^32^^,^^35^ Although TW might decrease with age,^4^ no differences were found between groups in this analyzes. Generally, TW is considered useful for differentiating atypical tympanogram shapes^32^^,^^35^ but seems to be redundant in pediatric tympanograms with well-defined Y_TM_ and TPP when there is no conjecture of middle ear alterations.

The mean Y_TM_1 kHz values obtained for the NB and 6‒8 m groups are very similar to those reported for infants in other publications, with no significant statistical difference between the groups.^4^^,^^8^^,^^18^^,^^22^^,^^27^ The values obtained are close to those suggested as normal standards for infants aged three to 26 weeks, distinguishing them from ears with conductive alterations: greater than 1.4 mmho and a positive tympanogram classification.^24^^,^^27^

Similarly, studies involving the same age groups reported Y_TM_ 226 Hz measures similar to those in this research^17^^,^^18^^,^^22^^,^^32^ and progressively higher values with increasing age were described.^17^ Keefe and colleagues^6^ showed a decrease in impedance during the first 12 months of life. Despite the notable increase in Y_TM_ 226 Hz with age, a statistically significant difference was evident only in the comparison between the 6‒8 m group and the older age groups (6‒8y and 9‒11y). The measures for the 3‒5y group appear to be a transition between those obtained for infants and those for children aged six years and older. An similar effect was previously reported and might be associated with more gradual changes in acoustic admittance throughout development.^4^^,^^22^

### Acoustic absorbance

The average absorbance indices in the NB group are consistent with experiments in neonates, with equipment similar or identical to that of this study, especially at frequencies from 0.25k to 0.6k Hz and from 6k to 8k Hz.^14^^,^^15^ Absorbance measurements were generally consistent with experiments in neonates for most sound frequencies, especially considering the 5 %‒95 % sample variation.^6^^,^^8^^,^^14^^,^^15^^,^^29^^,^^36^

The highest averages for NB were found between 2k and 8k Hz, and these results also align with studies that described low reflectance indices or high absorbance, especially around 1k to 3k Hz (with values higher than 0.6).^6^^,^^8^^,^^10^^,^^28^ High absorbance at 8k Hz was also reported in other studies with neonates.^4^^,^^14^^,^^15^ Measurements with the Titan device indicated lower average acoustic absorbance around 4k Hz compared to adjacent frequency ranges,^4^^,^^30^ an aspect not observed among the newborns in this research. However, low absorbance at 4k Hz was observed around two or three days of life,^30^ while the average observed in the NB group was 16-days. Longitudinal experiments that re-evaluated neonates after about a month^8^^,^^29^^,^^30^ showed an increase in absorbance at 4k Hz during this period. It is also noted that the variation in results in investigations encompasses the indices obtained for this frequency within the 5 %‒95 % percentiles of the sample.^4^

Unlike the experiment by Hunter and colleagues^33^ – which reported a significant increase in reflectance from birth to four years only at the frequency of 6k Hz – relevant changes in acoustic absorbance were observed throughout development in the present study. The effect of age was evident with a statistical difference for most sound frequencies. At low-frequencies (up to 0.6k Hz), the levels were significantly higher in the NB group compared to all others and, between 2k and 5k Hz, there was no statistical difference in the comparison between NB and 6‒8 m, but the difference occurred when comparing these with the other groups (3‒5y, 6‒8y and 9‒11y), showing a reduction in absorbance with increasing age.

The decrease in absorbance at low frequencies with a significant difference in the comparison between NB and 6‒8 m was also reported in other publications,^3^^,^^4^^,^^6^^,^^8^^,^^27^ especially after the first month.^3^^,^^4^^,^^6^^,^^8^^,^^27^, ^28^, ^29^ An increase of up to 30 % in reflectance indices up to 0.5k Hz has been described during this period.^6^ Similarly, the difference in average absorbance between 6‒8 m and NB was more expressive, the lower the frequency – at 0.5k Hz, it was 16 % lower, and at 0.25k Hz, 36 % lower. The absorbance indices of these frequencies, which were similar in the groups from 6‒8 m onwards, showed that this measurement should remain reduced throughout childhood. The structural and physiological transformation of the EAC after a few months of life would be responsible for reducing absorbance, admittance, and conductance at low frequencies.^4^^,^^6^^,^^8^^,^^28^

The mean absorbance levels in the 6‒8 m group were high from 1k Hz onwards and the results showed subtle differences from previous investigations of absorbance (or conversion from reflectance) for the age group.^3^^,^^4^^,^^6^^,^^8^^,^^28^^,^^33^ The 6‒8 m measurements were slightly lower at low frequencies (up to 1k Hz) and higher at high frequencies (from 2k to 6k Hz), but the 90 % range covers the cited studies, with results that can be considered similar. An experiment with the same device also showed high measurements in the range of 1.25k to 6k Hz in six-month-old infants; however, compared to newborns, they presented lower responses between 1k and 2k Hz and higher between 3k and 5k Hz, a range in which the indices would increase until 18-months.^4^ Other studies showed that up to six months, there is an increase in absorbance between 2k and 6k Hz^8^^,^^27^^,^^29^ and at the frequency of 4k Hz, this change was mentioned as more significant.^4^^,^^8^^,^^28^^,^^30^ In contrast, the average absorbance indices in the NB and 6‒8 m groups were similar from 2k to 6k Hz and, despite the high level at 4k Hz in the 6‒8 m group, as no reduced measurements were found at this frequency in NB, it was not possible to identify this difference.

One hypothesis for the high absorbance at medium and high-frequencies would be the increase in the tympanic cavity itself, not excessively at this age, but which would make the cavity acoustically more rigid^4^ at the same time that there is a global decrease in mass component in the ME, due to the clearance of the mesenchyme and fluids adhered to the ossicular chain, the development of the ossicles and the gradual increase tension in the joints.^3^^,^^4^^,^^6^^,^^8^^,^^27^ Therefore, the absorbance at high frequencies could remain robust in infants, as well as those obtained in the ears of newborns, probably due to the better conductance and, consequently, greater admittance of the ME. In contrast, there was a marked reduction in this measurement at 8k Hz in the 6‒8 m group and statistical significance in the multiple comparisons between NB and the other age groups. Although the difference at this frequency has been mentioned in other investigations,^4^^,^^30^ no consistent explanations for this fact have been presented.

In general, the results showed higher absorbance indices in the NB and 6‒8 m groups than in children aged three years and older – groups 3‒5y, 6‒8y and 9‒11y – whose measurements were quite similar to each other, being higher in the range of 2k to 6k Hz. Therefore, unlike the reduction in absorbance at low-frequencies, which should occur after the first month, at high-frequencies, it seems to occur only after the end of the first six months of life. The lower absorbance observed in the ears of children compared to infants was also reported in previous studies.^6^^,^^13^^,^^28^ The average indices obtained in the NB, 6‒8 m and 3‒5y groups were compatible with the normative reflectance data (converted) proposed by Hunter and colleagues^33^ for the same age groups – infants (up to five months) and children (between six and 47 months of age). An experiment with Titan on normal ears of children aged six months to three years^34^ showed high levels of absorbance in the frequency range from 1.5k to 6k Hz, and the values described were quite similar to those found in groups of similar age (6‒8 m and 3‒5y). At the same time, the results of the 3‒5y group were generally lower than those found at 16-months in the study by Myers and colleagues^37^ in this same frequency range, which suggests insignificant variations in low-frequencies between six months and one year, but significant changes in absorbance from 2k Hz onwards, which probably continue, but at a less accelerated progression, until approximately three years of age. This aspect may be related to additional important changes in the TM, ossicular joints, and tympanic cavity up to three years of age.^37^

Although increasing absorbance levels from 0.25k to 1.25k Hz and decreasing absorbance levels from 2k to 5k Hz were reported in a study of children aged four to 13 years,^16^ these results were not found to be statistically significant in multiple comparisons for the age groups 3‒5y, 6‒8y, and 9‒11y. However, as in another experiment that demonstrated greater absorbance at high frequencies in five-year-olds than in 12-year-olds and adults,^3^ the mean indices at frequencies from 2.5k to 4k Hz were higher in the 3‒5y and 6‒8y groups than in the 9‒11y groups. The present results showed that in the older age groups (6‒8 years and 9‒11 years), the absorbance indices were lower when compared to younger children and are already close to the values described for adults.^3^^,^^20^^,^^27^ These measurements were also close to the converted reflectance results of a previous study with children of the same age, being lower at low frequencies and higher from 2k Hz onwards.^20^ At frequencies from 2k to 6k Hz, the indices obtained, the results were slightly lower than other studies,^3^^,^^16^ but were consistent with the 5 %‒95 % ranges of the samples.

Accompanying body growth, the growth of the structures of the ME would accentuate the mass component in the conductive mechanism and, consequently, reduce the response to high-frequency sounds.^19^ In adults with normal hearing, progressively higher absorbance levels are found up to approximately 4k Hz, reducing again at frequencies above this.^6^^,^^14^ In the current study, high acoustic absorbance was also found at high frequencies at all ages, but in this case, up to 6k Hz. Thus, it is possible that the reduction above 4k Hz occurs only during adolescence.

It is considered that, at all ages, the range of 1k to 4k Hz shows the highest levels of absorbance, with better effectiveness in the direct transfer function through the ME. These levels tend to be reduced (there is higher reflectance) at frequencies below 1k Hz.^6^^,^^26^^,^^27^ This premise aligns with the present research, which found lower absorbance indices in the 0.25k‒0.8k Hz range across all age groups, while the high indices of 2.5k to 6k Hz in the entire sample indicated that, from birth to puberty, there is a natural effectiveness in the transfer of high-frequency sounds.

### Resonance frequency (RF)

In addition to acoustic absorbance measurements, some investigations on RF in different age groups were conducted using multifrequency tympanometry and WAI, determining which sounds are transmitted more efficiently, since the amplitude of TM displacement is greater at a corresponding frequency.^11^ The estimate of this frequency presents reasonable accuracy in predicting conductive alterations in different age groups and its clinical use in children does not require specific normative data by age, sex, ear or ethnicity.^20^

Some reports indicated lower RF, around 0.25k Hz in newborns, generally attributed to the external ear due to vibrations of the canal walls, and around 0.4k to 0.5k Hz in infants up to four months.^4^^,^^6^^,^^11^^,^^12^^,^^14^^,^^15^^,^^27^^,^^28^ These measures are compatible with the NB results, but not in 6‒8 m group. The increase in RF in the first months of life has also been described in other publications.^11^^,^^12^^,^^14^ A substantial increase in RF was observed with increasing age, up to three years, with no relevant differences after this period (groups 3‒5y, 6‒8y and 9‒11y). In all age groups, the means obtained were similar to those of another investigation carried out with the same equipment.^14^ The statistical difference occurred in the multiple comparisons between NB and the other age groups. Since there was no difference in the comparisons between 6‒8 m and the groups 3‒5y, 6‒8y and 9‒11y, the mean RF was notably lower in 6‒8 m than in 3‒5y, the results for 6‒8 m appear to be a transition between those found with Newborns (NB) and children. The middle ear RF should be higher, depending on the contribution of other vibrating elements, especially the TM, allowing greater amplitude of displacement at this frequency and more efficient transmission of sound energy.^11^^,^^15^ However, few differences in ME RF have been described during the first months of life and gain variations appear to be irrelevant, suggesting that structural changes are insufficient to modify resonance.^11^

Research carried out with children of different ages indicated ME RF with wide rates of variation in the samples, with the average being around 1.2 kHz in newborns and six-month-old infants,^4^^,^^11^^,^^27^ and around 0.8 kHz to 1 kHz in school-age children, with a progressive decrease with increasing age – 0.928 kHz from four to six years and 0.863 kHz between ten and thirteen years.^20^ In adults, it has also been described as around 0.8 kHz to 1 kHz,^38^ and can reach up to 2 kHz.^39^ As children grow, they would move from an efficient acoustic transmission system at high frequencies to increasingly lower frequencies,^21^ although the variations are still considered small.^15^^,^^20^

While a significant increase in external ear RF is expected during the first years of life, there should be a subtle and gradual reduction in the ME RF during development. This inversion would occur due to the increase in rigidity due to the greater length and ossification of the medial two-thirds of the EAC, reducing its cartilaginous thickness, which would increase the impedance for low-frequency sounds.^5^^,^^7^^,^^11^ Meanwhile, the tympanic cavity would change from small to larger dimensions, increasing volume and aeration, reducing the RF. The subtle changes related to ME RF could be explained by changes in the orientation and structure of the TM fibers, fusion of the tympanic ring, and tensioning of the ossicular joints, while increasing resistance (due to the increased air volume in the ME) and mass reactance (due to changes in the bone density of the ossicles).^12^

### Correlations between wideband acoustic immittance (WAI) measures

Although the MAE measurement cannot predict the sound energy transfer function, it is known that its growth in area and length strongly affects the input impedance components and the reflection coefficient, reducing absorbance at low-frequencies.^6^^,^^28^ In this study, correlation analyses showed an increase in RF associated with ECV growth, which in turn was related to a decrease in absorbance indices at frequencies up to 0.3k Hz and between 2k and 4k Hz. However, these correlations were not observed in each group, possibly due to the separation by age groups and the certain uniformity of ECV measurements among individuals in the same group. Furthermore, it was not possible to establish a direct relationship between ECV and WAI measurements that was not linked to the structural growth of the OE and increasing age.

In the 3‒5y, 6‒8y, and 9‒11y groups, there was a positive correlation between the Y_TM_ 226 Hz and absorbance measurements in the 0.25 kHz range. This correlation reinforces the concept that higher absorbance levels are associated with more efficient acoustic transmission in the same frequency range, increasing admittance through the TM and ME. However, this correlation was not observed in the 6‒8 m group. Similarly, in the NB and 6‒8 m groups, there was no correlation between absorbance at 1 kHz and Y_TM_ 1 kHz. It is possible that in this age group, it is still not possible to completely exclude the influence of the OE on acoustic admittance and absorbance measurements. Although significant changes in magnitude are expected throughout childhood, the admittance phase exhibits aspects similar to those of adults from four months onwards.

At low-frequencies, significant differences in magnitude and phase are observed in the first six months, but total admittance measurements, i.e., admittance gain and phase, vary little after this age. Since low-frequency admittances are usually high in newborns and younger infants, there is little variation after this age. In fact, the measurements obtained do not seem to exclude the mobility of the canal walls in the first months of life and could influence absorbance measurements at the same frequencies.^6^ Changes in the area and length of the EAC would explain most of the differences in susceptance up to 1.2k Hz and would alter admittance during the first year of life,^4^ but in the range from 1.25k to 2k Hz, total admittance measurements remain different throughout childhood compared to adults.^6^ Not only would the absorbance measurements and the magnitude of the admittance be high between 1k and 2.5k Hz in newborns, but also the smaller admittance angle in this frequency region would suggest proximity to the ME RF, indicating equal contribution from the mass and stiffness elements.^15^

In all groups from three years onwards, there was also a positive correlation between Y_TM_ 226 Hz and acoustic absorbance indices in the frequency range 0.8k‒1.25k Hz – the increase in absorbance was related to the increase in Y_TM_ 226 Hz, as described in another study.^28^ It is estimated that compliance, directly related to admittance, is essentially constant at frequencies close to 1k Hz and exhibits higher values with increasing age, but should be different for each sound frequency from that point on. With increasing age, the admittance level is expected to increase across the frequency range between 0.5k kHz and 8k Hz. In infants between one and six months, there is a peak near 0.4k Hz, while older infants and adults present progressively higher admittance with increasing age and relatively constant across the range from 0.25k to 1k Hz. At frequencies above 1k Hz, the results would be influenced by the mass of the ossicular chain, limiting the accuracy of obtaining an estimate of acoustic admittance.^6^ Absolute impedance levels in the ME are higher in infants, and this opposition to energy flow, attenuating input acoustic transfer for most frequencies, decreases as the ME develops. That is, the immaturity of the TM and ME is responsible for the lower compliance and higher resistance, increasing energy loss and generating lower transfer gain, especially in the 2k to 4k Hz range.^6^^,^^28^ Despite low reflectance levels (or high absorbance) in smaller ears, the efficiency in conducting energy through the ME is lower.^6^

The lack of significant correlation between Y_TM_ 226 Hz and RF in this study is possibly due to the uniformity of Y_TM_ 226 Hz results among most individuals in the same age group, reflecting adequate ME conditions, and the greater variation in RF in the OE.

Absorbance at medium and high-frequencies in fact, remains high during childhood and this aspect would reflect, although not completely, the acoustic transfer function through the middle ear. In infants' ears, for example, this higher absorbance is due to the small size of the tympanic cavity; at the same time, the acoustic admittance of the middle ear is lower than in adults. The growth-related reduction in high-frequency absorbance is attributed to the greater volume of the middle ear, which would make its RF lower and facilitate the transfer of low sounds. On the other hand, the smaller volume of the middle ear of younger children should increase the RF, being better for transferring high-frequency sounds.^6^^,^^9^ In smaller proportions, there is an increase in the footplate stapes and in the total area of the TM.

Therefore, it is expected that the greater mass component will cause a degradation of the middle ear response to high frequencies throughout childhood.^19^ However, the influence of middle ear volume and mass reactance on absorbance is still not fully understood and appears to depend on additional factors.

### Clinical implications

A publication that suggested cut-off criteria for infants up to six months old, with the aim of differentiating ears with normal hearing from those with conductive alterations, considered reflectance levels lower than 0.67, 0.69 and 0.64 for the frequencies of 1.25k, 1.6k and 2k Hz, respectively.^27^ Considering average absorbance levels at these frequencies, infants in the NB and 6‒8 m groups would meet the requirements for the expected standards. Likewise, the values obtained would be in accordance with high specificity criteria suggested for infants at the frequencies of 1k, 2k, 3k and 4k Hz in another study.^36^

Some considerations have been mentioned in studies with WAI, especially in relation to the high levels of absorbance for low-frequencies in newborns, which are usually attributed to the loss of energy resulting from the mobility of the EAC walls. Between 2k and 4k Hz, similar levels in comparison with other age groups would indicate that this OE compliance is not significant at high-frequencies.^6^ Increased physiological noise, test-retest variability, and probe sealing problems in the EAC are factors to consider when interpreting WAI measurements in infants.^6^^,^^10^^,^^33^^,^^38^ Some publications have suggested that absorbance values greater than 0.58 at low-frequencies or greater than 0.7 at more than six frequencies should be considered high or suggestive of energy escape.^6^^,^^10^ Likewise, it has been pointed out that in cases of reflectance indices lower than 0.3 at low-frequencies, replacement of the sealing olive and repositioning should be considered.^33^ At frequencies up to 0.3k Hz, absorbance measurements ≥ 0.7 in neonates or 0.3 in other age groups were reported as a criterion for discontinuing the test.^4^ Knowing that poor placement of the probe could cause energy leakage or differences in compliance in the cartilaginous portion of the neonatal canal,^10^^,^^33^ in this study, the care for adequate sealing during the examination of newborns was taken by inserting the probe as deeply as possible and changing the silicone olive and/or repositioning it (with new calibration in situ), when necessary. Even so, in some cases, high rates were found (> 0.7 in more than six and/or > 0.58 at low-frequencies), contradicting the parameters suggested by AlMakadma and colleagues.^10^ One way to ensure adequate sealing during data collection was to perform otoacoustic emissions tests after WAI measurements, with the same equipment and without changing the positioning of the probe, with results within normal standards. Although test-retest with reinsertion of the probe is a recommended method to avoid poor adjustment and minimize leaks,^3^^,^^6^^,^^38^ some experiments have shown that variations in repeated measurements in the same ears are smaller than those that can be attributed to different analysis systems, calibration methods, and inter-individual variability itself.^3^^,^^6^^,^^10^^,^^11^^,^^15^^,^^28^^,^^30^^,^^33^^,^^38^

Various studies with WAI have described a high variability in results, showing significant interindividual differences.^4^^,^^6^^,^^14^^,^^17^^,^^18^^,^^22^ The growth of the skull and, consequently, the tympanic cavity, improved pneumatization through the absorption of mesenchyme and elimination of amniotic fluid, as well as the development of the auditory tube, are believed to be related to the increase in admittance.^4^ These aspects would also reduce middle ear resistance.^4^^,^^6^ One of the limitations of this study may have been not differentiating the children evaluated according to their ethnicity, unlike other studies.^4^^,^^16^^,^^19^^,^^21^^,^^33^ This decision was associated with the difficulty in establishing hereditary patterns in a location with high rates of immigration and miscegenation throughout its history. The high ethnic diversity could also explain the great variability among individuals in the same age group in some measures. On the other hand, the heterogeneity of the sample may allow a more comprehensive understanding of child development, without the influence of subtle differences attributed to ethnicity or other demographic factors. Shahnaz and colleagues^21^ found differences not large enough in WAI measures related to ethnicity and body size that would not require the use of specific norms by ethnicity.

Another aspect to be considered is the number of participants. Considering some difficulties in testing infants and toddlers, such as agitation, speech, or crying, the sample size for 6‒8 m and 3‒5y was smaller than the other groups, because many children in these age groups did not cooperate or did not complete the research protocol. These challenges have also been mentioned by some authors.^8^^,^^28^^,^^33^ Other studies carried out in children with normal hearing at this age used samples of similar sizes.^14^^,^^27^^,^^28^^,^^30^^,^^33^^,^^34^ Although the sample size did not affect the statistical analysis, some age-related differences reported in larger experiments may not have been identified. However, beyond the scope of this article, it is worth noting that the results analyzed were confirmed by different statistical methods, and the analyses that were easiest to interpret were selected for this text.

Other difficulties in comparing studies could be attributed to procedures with different types of stimuli, in different equipment, probe formats, and calibration methods,^10^^,^^30^^,^^31^ making it necessary for clinicians to be aware of the influence of these criteria during instrumentation and when using normative data.^21^ Although the results are consistent with previous investigations conducted with individuals of different age groups and with different assessment techniques, the robust correlations between equivalent ear canal volume, low-frequency absorbance, and resonance frequency warrant further investigation.

Several structural and functional changes in the EE and EM contribute in different ways to a series of modifications in acoustic patterns and should influence how sounds are captured, filtered, and transferred by the auditory system. WAI has proven useful in obtaining information about acoustic properties in energy transmission. Throughout childhood, different admittance patterns were found related to the various WAI measures, such as ECV, Y_TM_, absorbance, and RF. Nevertheless, research on this topic does not always show a consensus. Some correlations remain unexplainable, and the changes related to each measure seem to occur in different ways and different proportions, depending on the frequency range.

WAI is still little used in clinical practice, probably due to the difficulty in quickly interpreting the results, and the existence of several normative criteria to be adopted depending on the device. However, there is a consensus regarding their efficacy in diagnosing conductive alterations with high sensitivity and specificity,^13^^,^^28^^,^^40^ if, above all, the age of the subject is considered. The importance of understanding this combination of maturational aspects and their influences on acoustic response properties may improve the analysis and interpretation of these measures, as well as other auditory tests. Although this research did not aim to analyze WAI results to identify conductive disorders, characterizing WAI in normal hearing children will allow for more accurate diagnoses, improving diagnostic accuracy in clinical settings, particularly for infants/children with ambiguous tympanometry results.

This research sought to investigate the properties related to the conduction of acoustic energy, such as admittance, absorbance, and resonance frequency, considering the influence of structural and functional changes on the auditory system development. Many differences were observed in the analyzed measurements, with evidence that some already occur during the first months of life, but this process extends throughout childhood.

## Conclusion

This study demonstrated that anatomical and physiological changes in the external ear and middle ear throughout childhood lead to changes in wideband acoustic immittance measurements, such that equivalent ear canal volume and acoustic admittance increase throughout childhood, while resonance frequency only increases in the first 3-years of life. The variable pattern of acoustic absorbance is likely representing the various structural changes throughout childhood that affect mass and stiffness components independently.

## Authors’ contributions

All authors contributed to the study's conception, design and correctness.

Ana Paula Bruner: Formal analysis; investigation; resources; data curation; writing-original draft; writing-review & editing; visualization; project administration.

Sumitrajit Dhar: Data curation; writing-review & editing; visualization; supervision; project administration.

Uzma Akhtar: Data curation; writing-review & editing; visualization; supervision.

Alessandra Spada Durante: Formal analysis; investigation; resources; data curation; writing-review & editing; visualization; supervision; project administration; funding acquisition.

Renata Mota Mamede Carvallo: Investigation; resources; writing-review & editing; visualization; supervision; project administration.

All authors read and approved the final version of the manuscript.

## Funding

Financial support for this study was provided by Fundação de Amparo à Pesquisa do Estado de São Paulo (FAPESP) Grants: 2014/15810-0 and 2017/25548-0, and *Coordenação de Aperfeiçoamento de Pessoal de Nível Superior – Brazil (CAPES)* ‒ Finance Code 001.

## Disclaimer statement

Study results have not been presented at conferences or symposia or published.

## Ethics approval

All methods were performed in accordance with the relevant guidelines and regulations. This study was approved by the Human Research Ethics Committee of Irmandade da Santa Casa de Misericórdia de São Paulo (number 5.571.490, CAAE 80,030,717.4.0000.5479).

## Declaration of competing interest

The authors declare no conflicts of interest.

## Data Availability

The clinical data used to support the findings of this study are available from the corresponding author upon request.
